# THE ROLE OF MUTUALITY ON THE QUALITY OF LIFE OF MID-LATE LIFE ADULTS WITH CHRONIC ILLNESS AND THEIR NURSES

**DOI:** 10.1093/geroni/igae098.2693

**Published:** 2024-12-31

**Authors:** Silvia Cilluffo, Karen Lyons, Barbara Bassola, Ercole Vellone, Gianluca Pucciarelli, Marco Clari, Valerio Dimonte, Maura Lusignani

**Affiliations:** University of Milan, Milan, Lombardia, Italy; Boston College, Chestnut Hill, Massachusetts, United States; ASST Great Metropolitan Hospital Niguarda, Milan, Lombardia, Italy; Tor Vergata University of Rome, Rome, Lazio, Italy; University of Rome Tor Vergata, Rome, Lazio, Italy; University of Torino, Torino, Piemonte, Italy; University of Turin, Torino, Piemonte, Italy; University of Milan, Milan, Lombardia, Italy

## Abstract

Mutuality between an adult with chronic illness and their nurse has received little investigation, despite the therapeutic nature of the relationship. Qualitative research by this team has led to conceptualizing nurse-patient mutuality as an interactive process involving developing rapport and going beyond, being a point of reference, and shared decision-making/care. To inform this area of research, our team developed and tested one of the only known measures of nurse-patient mutuality. This paper reports findings from our recent multi-center study that examined the association of nurse-patient mutuality on mental and physical quality of life (QoL) of nurses and mid-late-life adults with chronic illness. A total of 192 unique dyads (Mage= 64.5 for patients and Mage=41.8 for nurses) were sampled across Italian hospitals. Nurses and patients’ perceptions of their mutuality were positively correlated (p<.001Actor partner interdependence models found significant actor effects of mutuality on mental QoL for nurses and patients; the better the patient perceived mutuality with their nurse, the better their mental QoL (p<.001); the better the nurse perceived mutuality with their patient, the better their mental QoL (p=.002). However, a significant partner effect found the better the nurse perceived mutuality with the patient, the more likely the patient was to have poor mental QoL (p<.001). There were no significant associations with physical QoL. Discussion will focus on the need for further research on the role of nurse-patient mutuality on health outcomes and health behaviors and the complex role nurse engagement can have in patients experiencing poor mental health.

